# *Drosophila melanogaster* as a Model Organism of Brain Diseases

**DOI:** 10.3390/ijms10020407

**Published:** 2009-02-02

**Authors:** Astrid Jeibmann, Werner Paulus

**Affiliations:** Institute of Neuropathology, Domagkstraße 19, 48149 Münster, University Hospital Münster, Germany

**Keywords:** Fly, *drosophila*, brain disease

## Abstract

*Drosophila melanogaster* has been utilized to model human brain diseases. In most of these invertebrate transgenic models, some aspects of human disease are reproduced. Although investigation of rodent models has been of significant impact, invertebrate models offer a wide variety of experimental tools that can potentially address some of the outstanding questions underlying neurological disease. This review considers what has been gleaned from invertebrate models of neurodegenerative diseases, including Alzheimer’s disease, Parkinson’s disease, metabolic diseases such as Leigh disease, Niemann-Pick disease and ceroid lipofuscinoses, tumor syndromes such as neurofibromatosis and tuberous sclerosis, epilepsy as well as CNS injury. It is to be expected that genetic tools in *Drosophila* will reveal new pathways and interactions, which hopefully will result in molecular based therapy approaches.

## 1. Introduction

### 1.1. Drosophila as a brain disease model

Modelling human brain diseases in *Drosophila melanogaster* offers several advantages for investigation of molecular and cellular mechanisms underlying human disease. Short life span, large number of offspring, many genetic techniques, a well known anatomical situation and a wide variety of mutants are convenient characteristics of *Drosophila* as a model organism. Time and tissue specific inducible promoters are available [1–3]. Anatomic divergence between the fruit fly and humans is apparent, which may be not sufficient to recapitulate some morphological features of neurological disease but fundamental molecular pathways are highly conserved [4]. Functional analysis of human disease genes including high–throughput pharmacological screens as well as behavioral assays have become available in *Drosophila*.

Sequencing of the Drosophila genome revealed about 13.600 genes [5], being less than the human genome [6,7] and comparison of the genomes of humans and *Drosophila* reveals further differences concerning a higher microsatellite mutation rate in humans, whereas nucleotide diversity is more prominent in *Drosophila* [8] but this does not compromise *Drosophila* as a disease model.

One obvious disadvantage of using fly models is the risk that important pathogenetic factors are vertebrate-specific and may be ignored in invertebrate models. For example, immunological diseases such as multiple sclerosis cannot be modelled convincingly in *Drosophila melanogaster.* Furthermore, brain infarcts and brain hemorrhage cannot be analyzed in *Drosophila* because vessels are lacking and blood cells are mainly restricted to primitive hemocytes. However, most *Drosophila* models do reproduce some aspects of human diseases although one has to bear in mind that the differences between mammals and invertebrates represent potential drawbacks in modelling brain diseases.

### 1.2. Genetic tools in Drosophila

**Figure 1.**
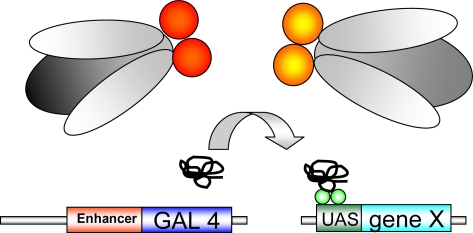
**GAL4-*UAS*** **system**: Transgenic flies expressing GAL4, a yeast transcriptional activator, are crossed against *UAS*-transgenic flies, carrying a gene of interest (“gene X”), inserted downstream of the *UAS* (upstream activating sequence;green balls). “Gene X” is activated in the offspring by crossing the two transgenic lines. The transgene is expressed in a time-/tissue dependent manner dependent on the selected GAL4-driver line.

In *Drosophila* a wide armamentarium of genetic tools is available. In a **reverse genetic approach** a candidate gene is tested for its potential functional role. One of the most important genetic systems used in reverse genetic approaches is the **GAL4/*UAS*-system** (see Figure 1.). Human proteins or *Drosophila* proteins can be expressed in a tissue and time dependent manner in the fly [9]. In most *Drosophila* disease models a human disease related transgene is inserted downstream of a *UAS* (upstream activating sequence) and can be expressed under the control of the yeast transcriptional activator GAL4. Absence of GAL4 results in inactivity of the transgene. After crossing flies carrying the transgene to flies expressing GAL4 under control of a cell- or tissue specific promoter, human protein expression is subsequently restricted to GAL4-expressing tissues. A large number of GAL4 driver lines including the glial promoter *repo (reversed polarity)*, the pan-neuronal promoter *elav (embryonal lethal, abnormal vision)* and the eye-specific promoter *GMR (Glass Multimer Reporter)* are available in *Drosophila*.

**Figure 2.**
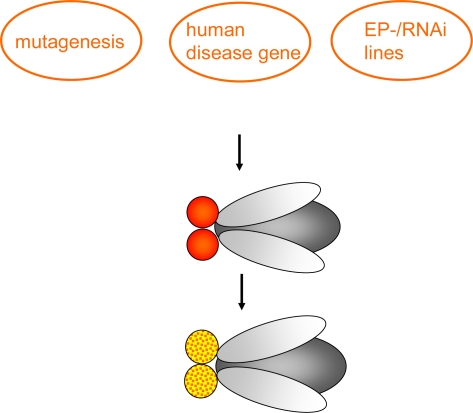
**Forward genetic screen:** Chemical mutagenesis (the chemical mutagen ethyl methane sulphonate (EMS) is common) or insertional mutagenesis techniques, having a disease-causing human gene, Enhancer-Promoter (EP)-element or RNAi construct placed under control of GAL4-responsive *UAS* sites are used to investigate the effect in specific tissues or the whole organism e.g. change of eye color (as indicated), reduced life-span, behavioral abnormalities or neuronal degeneration.

Compared to reverse genetics the **forward genetic approach** is an unbiased method – as to the function of a gene - for identification of genes based on phenotype. These approaches are usually conducted in one of two ways. On the one hand, a screen for mutations (chemical mutagenesis or insertional mutagenesis techniques (Enhancer-Promoter (EP)-elements and RNAi lines) (see Figure 2.) that reduce life-span, exhibit behavioral abnormalities or induce neuronal degeneration can be carried out. Several genes have been detected using these screens, among those the mutant *bubblegum* [10–12]. In principle, this approach can be useful to understand diseases whose genetic basis is undetermined yet [13].

**Figure 3.**
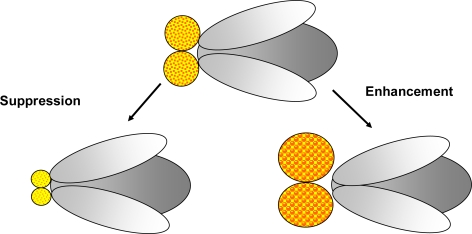
**Modifier screens:** A modifier screen is conducted in order to find genes that play a role in a process of interest. Random mutations created by mutagenesis or selected mutants already suspected to be involved in the pathway investigated as well as collections of Enhancer-Promoter (EP)-elements and RNAi stocks may be used to identify genes able to modify (enhance or suppress) the phenotype. In this figure suppression and enhancement of an eye phenotype is illustrated.

On the other hand, **modifier screens** can be used in order to find modifiers (suppressors/enhancers) of the disease phenotype in a mutant background (see Figure 3). Modifier genes are assumed to encode proteins that are related to pathways involved in the disease phenotype.

### 1.3. Drosophila CNS development

**Figure 4.**
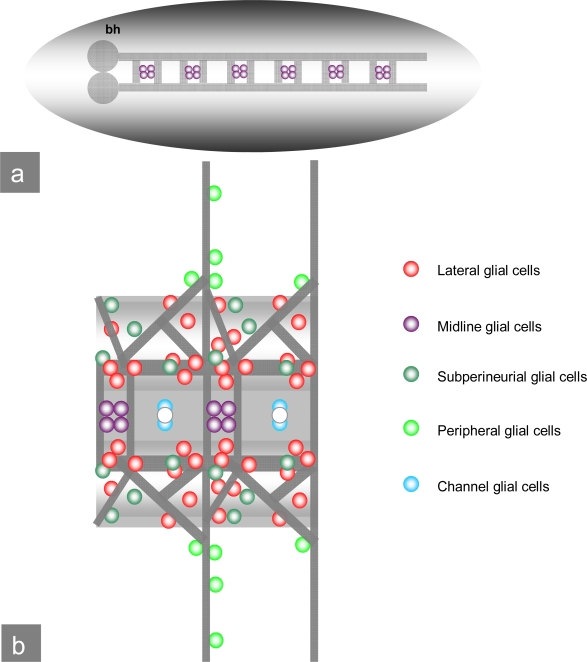
***Drosophila*** **embryonal CNS:** a) Schematic view of the *Drosophila* CNS and ventral nerve cord with brain hemispheres (bh), midline glial cells and commissures. b) Ventral view of the ventral nerve cord: Commissures, midline glial cells as well as subperineurial, peripheral and channel glial cells. (pictures modified after V. Hartenstein.)

*Drosophila melanogaster* is an insect undergoing metamorphosis, showing different developmental stages: embryo, larva, pupal stages and the adult fly. The central nervous system of the *Drosophila* **embryo** is composed of neurons and glial cells (Figure 4). The neurons build commissures in a close association with midline glial cells [14,15]. Glial cells in the *Drosophila* CNS can be classified either as midline glia or as lateral glia [16]. Surface glial cells form a continuous covering of the CNS and peripheral nerves. They comprise peripheral and exit glial cells, subperineurial glial cells, which enclose the CNS, and channel glial cells lining the channels lancing the ventral nerve cord [17].

**Figure 5.**
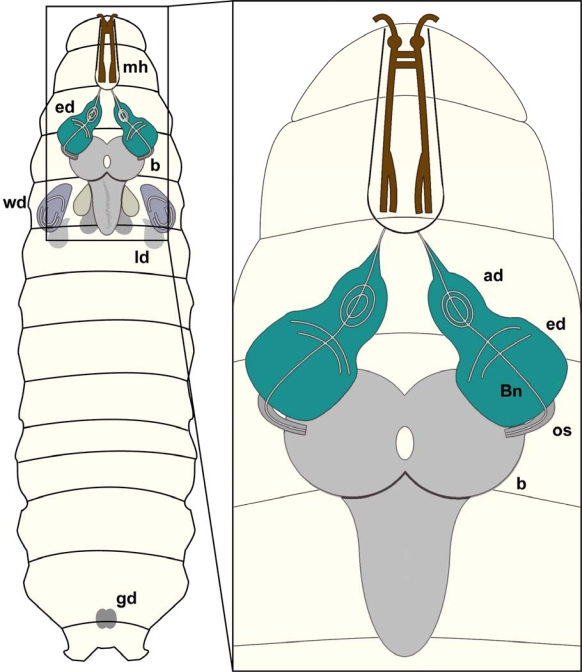
***Drosophila*** **larval CNS:**Schematic overview of a *Drosophila* larva showing brain (b), eye imaginal discs (ed), wing discs (wd), leg discs (ld), mouth hooks (mh) and gonads (gd).Magnification: Schematic view of brain hemispheres (b), eye imaginal discs (ed) as well as antennal discs (ad). The eye imaginal disc will form the adult compound eye whereas the antennal disc will develop into the antenna, the adult olfactory organ. The optic stalk (os) connects the brain hemispheres with the eye imaginal discs, whereas Bolwig nerve (Bn) constitutes the link of the larval brain to the larval photo receptors.

In *Drosophila,* various organs and anatomical structures arise from ten pairs of imaginal discs and the genital disc. The brain of the *Drosophila* **larva** is composed of two hemispheres and the subesophageal ganglion (Figure 5).

**Figure 6.**
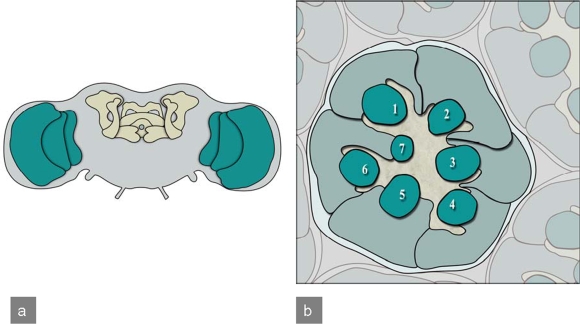
***Drosophila*** **adult CNS and compound eye:** a) Frontal view of the adult *Drosophila* brain. Highlighted are mushroom bodies and the central complex (yellow) as well as the optic lobes (green).b) Tangential section of a *Drosophila* compound eye reveals the highly stereotyped arrangement of ommatidia. Here the organization of photoreceptors for a single ommatidium is illustrated. Numbers 1–7 indicate the light-sensing organelles of the photoreceptors, called rhabdomeres. Photoreceptors R1-R6 have a longer rhabdomere and represent the outer photoreceptors, as they surround the inner rhabdomeres R7 and R8. In this section only R7 is visible as R8 sits directly underneath.

In brain disease models, brain and the complex eye of **adult** *Drosophila* are often used. Especially the mushroom bodies (Figure 6), association areas necessary for olfactory learning and memory [18], composed of about 2,500 Kenyon cells, neurons with small, densely packed cell bodies [19], are relevant in brain disease models. Additionally, the fly visual system is also often used as a model system in brain research [20,21] as it is less complex than the brain, formed by neurons which develop in a very stereotyped manner and can be conveniently investigated. In particular a “rough eye” phenotype caused by pathological processes can be easily studied, enabling screening for genes related to the process of interest. Eye development always begins with the differentiation of R8 photoreceptor neurons at uniformly spaced positions. These neurons signal to neighboring cells to develop ommatidia (unit eyes). Sequential differentiation of the other seven photoreceptor types (R1-R7) follows afterwards [22]. Each R8 neuron recruits one cell of each type, such that seven photoreceptors cluster around each R8 neuron [23] (Figure 6).

## 2. Neurodegeneration

### 2.1. Alzheimer’s Disease

Alzheimer’s disease (AD) is the most common neurodegenerative disease characterized by extracellular accumulation of Aß peptide in senile plaques and intracellular accumulation of hyperphosphorylated tau as neurofibrillary tangles and neuropil threads [24,25]. Familial AD, which makes up only less than 1% of cases, is caused by mutations of genes encoding amyloid precursor protein (APP), presenilin 1 and presenilin 2 [26].

Because not all components of APP proteolytic processing are conserved, modelling AD in *Drosophila* is quite challenging. The *Drosophila* APP homolog *(dAPPL)* does not contain the Aß domain and cannot be cleaved in flies [27,28]. Studies in *Drosophila* overexpressing wildtype human *APP* (*hAPP695*), *hAPP695* with pathogenic mutations (*hAPP*-*Swedish*), full length *hAPP* with N-terminal myc tag as well as a construct comprising a signal peptide, ßA4 region and a C-terminal myc tag demonstrated γ-secretase activity in flies [29]. BACE activity is not present in *Drosophila*, whereas γ-secretase, presenilins and nicastrin are conserved [30–32].

Expression of human Aß40 and Aß42 in the fly brain caused age-dependent learning defects but only Aß42 led to formation of diffuse amyloid deposits in the Kenyon cell region positive in the thioflavin S staining but in transmission electron microscopy without clear amyloid fibril structure, neurodegeneration, and decline of locomotor functions [33] Expression of Aß42 in the CNS was found to cause short-term memory impairment [34]. Expression of human Aß42 in the eye caused degeneration of the retina, which could be suppressed by neprilysin overexpression [35]. In another approach human *BACE* and *hAPP* were expressed singly or in combination in fly eyes using *gmr-*GAL4. Expression of *hAPP* alone or in combination with *BACE* resulted in degeneration of retinal photoreceptors and age-dependent plaque formation, which could be enhanced by coexpression of the *Drosophila presenilin* (*UAS-DPsn*) containing a point mutation corresponding to the FAD mutant L235P. Amyloid deposits were positive for thioflavin S, ß-amyloid as well as Aß42 detecting antibodies in flies expressing *hAPP* and *DPsnL235P* or *hAPP*, *BACE* and *DPsnL235P.* Electron microscopy revealed “star-like” deposits in the retina, neuropil regions and optic ganglia [36]. Taken together in this study a sequence of neuronal degeneration followed by plaque formation is described, which does not recapitulate the chronological sequence of AD in humans. Secondly gender differences could be detected with respect to plaque formation being more severe in male than in female fliese, a fact which is not seen in human AD. On the other hand, age dependent progression of neurodegeneration and plaque formation is detected, immunohistochemical staining profile and EM ultrastructure with star-like formation resembles human AD plaques [37].

Further studies using deletion and overexpression of *dAPPL,* expression of *hAPP695* and of *hAPP* mutants (*hAPP-London* and *hAPP-Swedish*) in larval motor neurons found accumulation of organelles along axonal tracts [38], suiting well to axonal transport disturbances seen in transgenic mouse models [39]. The neurofibrillary, tau associated pathology is lacking in *Drosophila* Alzheimer models, a fact that has also to be considered in the majority of mouse Alzheimer models [40].

Taken together, the fly can be used to study some molecular aspects of Alzheimer disease although the disease models remain more artificial than rodent models.

### 2.2. Tauopathies

Neurofibrillary tangles (NFT), neuritic plaques and neuropil threads composed of hyperphosphorylated tau forming intraneuronal inclusions are characteristic of AD, but tau inclusions are also associated with other neurodegenerative diseases such as Pick’s disease, corticobasal degeneration, argyrophilic grain disease, progressive supranuclear palsy and fronto-temporal lobar degeneration (FTLD) [41] characterized by diverse clinico-pathological phenotypes from aphasia to dementia syndromes [42].

*Drosophila* has one *tau* gene which is expressed in neurons and localized in axonal processes [43]. By expressing human wildtype *tau* and FTLD-linked mutant forms of tau (R406W, V337M) in cholinergic neurons a fly model of tauopathies was established showing adult-onset progressive neurodegeneration with vacuolization and reduced life span, particularly in R406W transgenic flies. Abnormal tau protein accumulated but NFT could not be detected [44]. When wildtype human tau (4R isoform) was coexpressed with the fly homolog of *GSK-3β (shaggy),* presumably involved in hyperphosphorylation of tau, neurodegeneration was enhanced and insoluble intraneuronal NFT formation, positive for AT100 antibody was observed. Ultrastructurally filamentous structures, including paired helical filaments with a characteristic periodicity as well as straight filaments were detected [45]. Antibody AT100 stains both intracellular NFT and extracellular NFT [46]. As a consequence of neuronal expression in this *Drosophila* model extracellular NFTs cannot be dectected in these flies. Ultrastructural findings in these flies are similar to paired helical filaments and straight tubules found in a wide range tauopathies [47] as well as detergent-resistance, another characteric of tau accumulations [42]. Toxicity of wildtype *dtau* and *htau* under control of *gmr*GAL4 was dosage dependent, and coexpression of these genes with known modifiers of *tau* differed in approximately half of the investigated *Drosophila* lines. Comparing *dtau* and *htau* in modifier screens in *Drosophila* model may clarify the degree of functional homology [48].

Antiapoptotic genes (*p35, DIAP1* and *DIAP2*) were shown to reduce tau toxicity [45]. Overexpression of wildtype human *tau* in motor neurons disrupted axonal transport leading to locomotor phenotypes. Again, these could be enhanced by coexpression of *GSK-3β* and reversed by GSK-3β inhibitors lithium and AR-A014418 [49]. Modifier screens revealed a number of kinases, among those *par-1* [48,50,51] to be involved in tau related neurodegeneration.

Thus, *Drosophila* tau models replicate several important features of human tauopathies, including tau hyperphosphorylation, accumulation, NFT formation and neuronal degeneration.

### 2.3. Parkinson’s Disease

Parkinson’s disease (PD) is a movement disorder showing resting tremor, rigidity, akinesia and postural instability, which mostly occurs sporadically, while hereditary cases represent less than 10% of patients [52]. Familial PD cases have been related with mutations, duplications and triplications in *SNCA* (α-synuclein)/*PARK1*, parkin/*PARK2*, *UCHL-1* (ubiquitin carboxy-terminal hydrolase L1)/*PARK5*, DJ-1/*PARK7*, PINK-1 (PTEN-induced putative kinase)/*PARK6*, LRRK2/*PARK8,* ATP13A2 (p-type ATPase)/*PARK9*, and HTRA2 (HtrA serine peptidase 2)/*PARK13*. Other *PARK* loci have been identified, but the mutated gene is unknown [52]. Neuropathological hallmarks are loss of dopaminergic nigrostriatal neurons and typically accumulation of α-synuclein in cytoplasmic inclusions called Lewy bodies (LB) and Lewy neurites [52].

To create a fly model of PD, wildtype human α-synuclein and two familial mutant forms (A30P and A53T) were expressed in dopaminergic neurons. Expression of α-synuclein led to age-related loss of dopaminergic neurons, LB-like accumulations and behavioral deficits. In flies expressing mutant α-synuclein in a pan-neuronal pattern α-synuclein inclusions were also found in non-dopaminergic neuronal cell bodies like in human PD brains [53]. LB-like structures in the fly stained positive for α-synuclein and were ultrastructurally composed of filaments with granular material similar to human Lewy bodies. [53,54]. l-DOPA, a drug used to treat PD patients, could suppress behavioral defects [55].

Mutations of *PARK2* encoding parkin are linked to autosomal recessive, juvenile onset parkinsonism [52]. Loss of function mutations of *Drosophila parkin* increased sensitivity to oxidative stress [56,57] and showed pathological mitochondrial structure although no dopaminergic neurodegeneration could be observed. Other studies suggested *parkin* protection against α-synuclein damage to dopaminergic neurons [58]. In another model expression of two mutant forms of human parkin, Q311X and T249R in dopaminergic and serotoninergic neurons under control of the *ddc*-GAL4 driver, selectively caused degeneration of dopaminergic neurons and progressive locomotor dysfunction [59].

In humans mutations in PINK-1 lead to early-onset autosomal PD [52]. Loss-of-function mutations of the *Drosophila PINK1* homolog show dopaminergic neuronal degeneration, flight muscle degeneration, locomotor defects and mitochondrial defects. Interestingly, expression of parkin could ameliorate *PINK1* phenotypes [60].

Mutations of DJ-1 lead to an early onset autosomal recessive variant of PD [52]. Loss of the two DJ-1 homologs in *Drosophila DJ-1α* and *DJ-1β* was investigated and flies with deletions of *DJ-1 β* and *DJ-1β* were viable, fertile and showed normal lifespan. Interestingly these flies were selectively sensitive to toxins like paraquat and rotenone, linked to sporadic PD in humans [61]. Loss of function of *DJ-1β* led to locomotor deficits without loss of dopaminergic neurons [62].

Mutations of LRRK2 cause a late onset autosomal-dominant form of PD [52]. In flies expressing wildtype fly *LRRK* and the Arg1069Cys mutation corresponding to pathogenic Arg1441Cys mutation in LRRK2 associated with PD [63] under control of various GAL4 driver lines inducing whole body, muscle, eye and dopaminergic neuron specific expression did not show defects, but *LRRK* loss-of-function mutants showed decreased locomotor activity and reduction of dopaminergic neurons [64]. A second gain-of-function LRRK2-PD model was established using human LRRK2 and LRRK2-G2019S, another mutation associated with PD [65,66]. Expression of wildtype and mutant human *LRRK2* in photoreceptor cells by *gmr-*GAL4 led to neuronal degeneration and expression in dopaminergic neurons resulted in selective loss of those neurons, locomotor dysfunction and reduction of life span [67].

Rotenone treatment of wildtype flies led to loss of dopaminergic neurons and locomotor defects [68]. Treatment of wildtype flies with paraquat led to impaired climbing capability and decreased survival which could be restored by cannabinoid receptor agonists (CP55,940) and a specific inhibitor of stress responsive Jun-N-terminal kinase signalling (SP600125) to different extent. [69].

Taken together, several genes involved in PD as well as PD models utilizing toxins have been investigated in the fly. To a remarkable extent neuropathological hallmarks could be modeled in *Drosophila.* Interdependence of different genes can be suitably investigated in this organism and is expected to influence understanding of the disease.

### 2.4. Prion Diseases

Prion diseases are rare fatal neurological diseases of genetic or infectious origin, but most often occur sporadically. In humans, sporadic (sCJD), familial (fCJD) and variant (vCJD) Creutzfeldt-Jakob disease, Gerstmann-Sträussler-Scheinker disease (GSS), fatal familial insomnia (FFI) and kuru are known [70]. Prion diseases are induced by misfolding of prion protein PrP^C^ into one of several pathogenic isoforms [70,71].

GSS disease, one of the inherited prion diseases can be caused by some mutations of the prion protein gene, but most commonly by a point mutation at codon 102 and methionine at position 129 [72]. Histologically in GSS, widespread, large, multi-centric amyloid plaques with a dense core encircled by satellite globules located predominantly in the cerebral cortex and cerebellum prevail, positively stained by PrP antibodies, accompanied by white matter degeneration and neuronal loss, spongiform changes, gliosis and NFTs. Ultrastructurally amyloid plaques consist of radiating bundles of curvilinear filaments without definite periodicity [73].

The first effort to model a prion disease in *Drosophila,* an organism which does not have a prion gene, involved the expression of Syrian hamster prion protein under control of a HSP70 promoter [74]. Transgenic flies produced full-length prion protein on heatshock. No phenotypes were observed and the expressed prion protein never appeared to achieve the pathological protease-resistant form [74]. In the only prion fly model so far, a mouse prion protein with a proline to leucine mutation (P101L), homologous to a human mutation (P102L) causing GSS was used [75], leading to locomotor dysfunction, reduced life span, progressive vacuolar pathology and PrP inclusion bodies. Increase in proteinase K resistance (a specific feature of PrP^Sc^ diseases) was not seen, but biochemical analysis suggested that PrP is aberrantly folded. Transmissibility to other flies and ultrastructure of inclusions were not investigated [75].

This model can be useful with regard to understanding the pathobiology of P102L. GSS is a transmissible disease characterized mainly by proteinase K-resistant PrP positive plaques, amyloidosis and spongiform degeneration whereas the fly model shows vacuolation and inclusions rather than plaque formation, but not proteinase K resistance. Although neuropathology and biochemistry appear to be different in these two species, modifier screens may be of interest to better understand selected molecular pathogenetic pathways.

### 2.5. Polyglutamine Disorders

Several hereditary neurodegenerative diseases known as polyQ diseases result from CAG repeat expansion within the respective disease genes [76]. These include Huntington disease, X-linked spinobulbar muscular atrophy (SBMA, Kennedy disease) and the spinocerebellar ataxias SCA1, SCA2, SCA3 (also known as Machado-Joseph disease), SCA6, SCA7 and SCA17 as well as dentatorubral pallidoluysian atrophy (DRPLA).

#### 2.5.1. Huntington’s disease

Huntington’s disease (HD) is an autosomal dominant illness with psychiatric, cognitive and motor symptoms, in particular chorea, caused by unstable expansion of CAG repeats within the coding region of the gene *IT15* on 4p16.3. The disease occurs when more than 37 polyQ repeats are present [76]. In the majority of cases atrophy of frontal lobes and bilateral atrophy of the striatum are seen. In the caudate nucleus neuronal loss and reactive astrocytosis are detected. Ubiquinated, neuronal nuclear inclusions can be found [76].

Directed expression of exon 1 of the human *IT15* gene containing 2, 75 or 120 polyglutamine repeats in *Drosophila* causes late-onset progressive neurodegeneration dependent on repeat-length as it is typical of human HD. Huntingtin protein accumulates in the nucleus and could be stained with anti-huntingtin antibodies but does not form HD-specific inclusions. Nuclei were found to have spherical particles indistinguishable from virus-like particles induced by a transposable element found in several fly strains [77].

Knock-down of the *Drosophila* homolog of *IT15* gene *huntingtin (htt)* causes axonal transport defects, showing a phenotype similar to overexpression of the human HD gene [78], indicating that *Drosophila* htt is required for normal axonal transport.

In a recent study repeat instability of an HD *Httexon1Q93* transgene could be demonstrated, a key aspect of polyQ diseases [79]. Modifier screens comparing a *Drosophila* Huntington model and SCA1 revealed a number of genes related to both diseases but others even showed opposite effects in the different disease models [80]. The question of how polyglutamine expansion mediates toxicity was addressed using a yeast two-hybrid screen in a *Drosophila* SCA1 model using several human ATXN1 constructs with wildtype (30Q) or expanded (82 Q) polyglutamine tracts and varying phosphorylation status at serine 776 (S776). The screen showed that polyQ expansion favors formation of a protein complex containing RBM17 (RNA-binding motif protein 17) and attenuates formation and function of a protein complex containing the HMG-box protein capicua (CIC), providing insight into molecular pathogenesis of SCA presumably representative for other polyglutamine diseases [81].

#### 2.5.2. Spinocerebellar ataxia type 3 (Machado-Joseph disease; SCA3)

SCA3 patients present with cerebellar ataxia, pyramidal signs, extrapyramidal symptoms, peripheral neuropathy, nystagmus, eyelid retraction and facial fasciculation [82]. This dominantly inherited disorder is linked to an unstable CAG repeat *ataxin-3* gene on chromosome 14q32.1 [82]. Pathologically, brain atrophy, atrophy of the brain stem and spinal cord, as well as depigmentation of substantia nigra are found. Anterior horns show severe neuronal loss which leads to atrophy of anterior spinal roots and skeletal muscles. Nuclear inclusions are found in almost all brain regions but Purkinje cells are spared. Inclusions are positive for ubiquitin and ultrastructurally are non-membrane bound, containing a mixture of granular and filamentous structures.

The first model of a polyQ disease in *Drosophila* was made by expressing the C-terminally truncated domain of the pathogenic human protein (SCA3tr-Q78) and the control protein (SCA3tr-Q27). Phenotypes were only observed in fly strains expressing the longer polyglutamine repeat [83]. Expression of *SCA3tr-Q78* in the eye using *gmr-*GAL4 caused loss of pigmentation, destruction of the retina and nuclear inclusions. The fly SCA3 model is promising because several key features of human disease are present, including neuronal degeneration, nuclear inclusions and trinucleotide repeat instability.

Partial rescue of fly pathology could be achieved by co-expression of the antiapoptotic protein p35 [83] or HSP 70 [84]. Furthermore, suppression by HSP70 can be synergistically enhanced by co-expression of *DnaJ-like-1 (DnaJ-1),* a homolog of the HSP40 chaperone protein [85]. Pathogenicity of the truncated ataxin-3 protein is more severe than that of the full-length protein, due at least in part to the protective nature of functional domains of the normal protein [86]. A recent modifier screen for *SCA3trQ78* toxicity yielded 17 suppressor and one enhancer gene which belonged mainly to chaperones and ubiquitin-pathway components and were considered to some extent to play a role in protein misfolding in general [87]. Coexpression of a the *Drosophila* homolog of *ATAXN2, using a UAS*-*Atx2* construct under control of *gmr-*GAL4 together with the pathogenetic human *Atx3 UAS-SCA3trQ78* strongly enhanced eye degeneration and increased inclusion formation [88]. Understanding of the interdependence of different SCA related genes and RNA-based trinucleotide repeat expansion diseases may be crucial with respect to future therapeutic advances.

As one important factor seen in polyQ expansion diseases is trinucleotide repeat instability, this was investigated in flies. In a SCA3 model using *SCA3tr-Q78* instability was shown to be enhanced by reduction of cAMP response element-binding protein (CREB)-binding protein (CBP), a key regulator in dna repair, whereas treatment with histone deacetylase (HDAC) inhibitors seemed to protect against repeat instability [79].

#### 2.5.3. X-linked spinobulbar muscular atrophy (SBMA; Kennedy Disease)

SBMA is a rare X-linked progressive motor neuronopathy caused by CAG repeat expansion in the first exon of the androgen receptor (AR) gene on Xq13–21 [82]. It is characterized by muscle cramps, proximal muscle weakness, atrophy and fasciculations as well as endocrine abnormalities like gynecomastia and testicular atrophy [82].

Pathologically, reduced numbers of motor neurons in the spinal anterior horns, facial and hypoglossal nuclei prevail and intranuclear inclusions consisting of granular dense aggregates of AR-positive materials are detected in the remaining motor neurons as well as in the skin, testis and other organs [82].

A SBMA fly model expressing mutant hAR (polyQ 52), a pathogenic form of the androgen receptor gene under control of *gmr-*GAL4 showed ligand-dependent neurodegeneration, encompassing marked disruption of the eye, reduced ommatidia numbers and loss of pigmentation with thinned retinas [89]. Although neuronal loss is represented in this model, other features of the human disease are not encountered, such as inclusions and involvement of other organs like skin and testis. Nuclear localization of the mutant protein was an obligate requirement for toxicity [89]. In a modifier screen *hoi-polloi (hoip)* gene, involved in small nucleolar RNA-protein (snoRNP) complexes which play a role in ribosomal RNA processing was identified as an enhancer of neurodegeneration, linking polyQ toxicity to dysregulation of translational activity [90].

#### 2.5.4. Non coding trinucleotide repeat diseases

Non coding trinucleotide repeat diseases characterized by expansion of trinucleotide repeats comprising CGG, CTG, CAG, GCC and GAA within the 5’ or 3’ untranslated region (UTR) or introns of a gene [91] cause diseases like spinal muscular atrophy, SCA 8 and SCA12. Repeat expansion within the noncoding region is made responsible for loss of function of the disease gene or gain of function of the disease-associated mRNA or both, leading to neuronal degeneration.

#### 2.5.5. Spinocerebellar ataxia type 8 (SCA8)

SCA8 is characterized by progressive gait and limb ataxia, dysarthria and nystagmus at variable ages of onset [82] caused by expansion of a CTG repeat in the 3’ UTR of the *SCA8* gene on chromosome 13q21 [92]. Histologically, severe loss of Purkinje cells is a predominant finding but neuronal loss may also occur in the inferior olivary nucleus and substantia nigra. Surviving Purkinje cells are atrophic and show somatic sprouts. Intranuclear inclusions positive for polyglutamine and ubiquitin positive inclusions are found in Purkinje, dentate and medullary neurons [82].

A *Drosophila* model using human SCA8 cDNA placed under control of a *UAS* element using a wildtype (SCA8[CTG9]) and CTG expanded (SCA8[CTG112]) form has been established [93]. Expression of both constructs in the eye under *gmr-*GAL4 control causes a rough eye phenotype and progressive degeneration of photoreceptor cells. Inclusions were not found. In a modifier screen mRNA binding proteins (*staufen (stau), muscleblind (mbl) and split ends (spen)*) have been identified but their functional role in RNA induced toxicity is still unclear [93]. Although fly neuropathology does not convincingly recapitulate human disease, the model may lead to better understanding of fundamental aspects of RNA induced toxicity.

#### 2.5.6. Spinal muscular atrophy (SMA)

Spinal muscular atrophies (SMAs) are genetically heterogeneous inherited diseases with progressive muscle degeneration caused by loss of spinal motorneurons. [94]. Nearly all SMA patients show alterations of the survival of motor neuron gene 1 *(SMN1)* on 5q13, which leads to loss of its protein product SMN [95]. The SMN protein is found in the nucleus and cytoplasm of all cells, but most abundantly in motorneurons. Histologically, symmetric loss of motor neurons as well as neurogenic atrophy of muscles is characteristic [94].

Ectopic expression of the human *SMN1* in *Drosophila* leads to pupal lethality [96]. Abnormal larval locomotion in homozygous mutants for the *Drosophila survival motor neuron (Smn)* homolog [97] could be explained by defect larval neuro-muscular junctions which showed disorganized and enlarged synaptic boutons as it is seen in SMA patients. No obvious muscular or neuronal defects were seen immunohistochemically. In a genetic screen using an SMN allele encoding a point mutation seen in SMA patients 27 modifiers of *Smn* lethality have been found, including some (*wishful thinking (wit)* and *Fmr1)* that have been shown previously to function at the NMJ and others which were not associated with NMJ function before [98]. Synapses at the neuromuscular junction are glutamatergic, providing at least some similarities to the spinal cord synapse which is affected in SMA.

### 2.6. Amyotrophic lateral sclerosis

Amyotrophic lateral sclerosis (ALS) is characterized by upper and lower motor neuron defects including brisk reflexes, spasticity and pathological reflexes, fasciculations and weakness [99]. More than 90% of ALS cases are sporadic, but the disease can be inherited as an autosomal or X-linked familial condition. 15% of familial cases are caused by mutations in the gene encoding the enzyme superoxide dismutase-1 *(SOD1)* [100]. Histologically, ALS is characterized by selective loss of motor neurons in the brain and spinal cord. ALS is characterized by ubiquitin-positive inclusions (“skein-like”, “Lewy body-like” or a combination of the two), small eosinophilic inclusions in spinal motor neurons (“Bunina bodies”), hyaline inclusions [101] as well as TDP-43 positive filamentous inclusions [102].

Expression of *hSOD1* (WT and A4V and G85R mutants) in motor neurons using the D42 motor neuron driver led to progressive motor dysfunction, whereas expression of *Drosophila Superoxide dismutase (Sod)* using the same driver line did not show an effect [103]. Loss of climbing ability was not restricted to mutant forms of *hSOD1* but was also observed in wildtype *hSOD1*. Hypothetically hSOD1 may be recognized as toxic mutant form of SOD1 in *Drosophila* and therefore led to this effect. Interestingly neither loss of motor neurons nor retinal degeneration was observed. *HSOD1* forms intracellular inclusions but solubility was not altered. [103].

A recent publication of a *Drosophila* model of ALS8 [104], established by using the respective *Drosophila* mutation (*dVAP33A*) of the human disease causing dominant mutation of VABP (vesicle-associated membrane protein (VAMP)-associated membrane protein B) showed that the the mutant protein aggregates, is ubiquitinated and recruits wild type protein into aggregates. Furthermore a new mechanism underlying ALS pathogenesis at the neuromuscular junction involving BMP signaling pathways could be identified [104]. In *Drosophila* synapses at the neuromuscular junction are glutamatergic, which provides similarities to the spinal cord synapse being of central interest in ALS in humans [104].

Earlier attempts to model ALS yielded confusing results as expression of *hSOD1* transgene and human mutated Gly41-to-Ser *SOD* in motorneurons by *D42*-GAL4 led to increased longevity and could rescue the lifespan of a *dSod* null mutant with shortened lifespan [105,106].

Taken together, publications about ALS in *Drosophila* did not show motor neuron loss, the observed intracellular inclusions did not reflect characteristics of human ALS inclusions, and investigations focussed on the neuromuscular junction. But despite of apparent discrepancies, these results will constitute the basis of modifier screens and are expected to contribute to further understanding of ALS pathogenesis.

## 3. Metabolic disorders

Up to now in *Drosophila* a number of metabolic brain disease models for lysosomal storage diseases, mitochondrial diseases and peroxisomal diseases exist. Three of them will be presented.

### 3.1. Leigh Disease

Leigh disease is a progressive mitochondrial encephalopathy characterized by psychomotor delay starting during the first months of life. Life expectancy is 1–4 years. Optic atrophy is also frequently observed [107]. The disease is caused by inherited mutations in both nuclear- and mitochondrial-encoded genes involved in energy metabolism, including mitochondrial respiratory chain complexes I, II, III, IV, and V, crucial to electron transport [108].

Macroscopically, symmetric necrotizing lesions in subcortical areas of the CNS (brain stem, cerebellum, diencephalon and corpus striatum), which are associated with spongiosis, demyelination and astrocytosis, are observed [109]. Ultrastructurally mitochondrial inclusions are found in the neurons of the brain, as well as increase of mitochondrial antigens in neurons, vessels, plexus and astrocytes [110].

In the fly photoreceptor cells mutant for *Succinate dehydrogenase* (*Sdh)* develop completely normally and innervate the appropriate synaptic partners. After some time receptor cells degenerate, progressively losing expression of synaptic markers, and undergoing extensive morphological changes [111]. Thus this model may capture some elements of the human disease.

Another model of Leigh disease was established using downregulation of *Surfeit1 (Surf1)* showing a number of behavioral and electrophysiological abnormalities including reduced photoresponsiveness, reduced locomotor speed and impaired optomotor response as well as abnormal electroretinograms with different driver lines [112]. *Surf1* downregulation driven by an *actin*-GAL4 line showed an underdeveloped CNS, while CNS wide silencing of *Surf1* driven by *elav*-GAL4 led to prolonged lifespan, normal CNS development, slight impairment of locomotor activity and photobehavior; histochemical reaction to COX was reduced in the optic lobes.

Ultrastructural investigation of the body wall muscle fibers in *actin*-GAL4 driven flies showed larger mitochondria, different distribution and morphological alterations [112], while other key features of human brain pathology were not observed.

Remarkably, mutant flies exhibited increased longevity which does not reflect the course of human disease.

### 3.2. Nieman-Pick-Disease

Niemann-Pick disease represents a group of lysosomal lipid storage disorders. Niemann-Pick type C (NPC), an autosomal-recessive disease, shows a wide spectrum of phenotypes with variable begin from perinatal period to adult age. Major neurological symptoms include cerebellar ataxia, dysarthria, dysphagia, seizures and progressive dementia [113]. NPC is characterized by accumulation of cholesterol, glycospingolipids and other lipids.

A defect of organelle trafficking and a failure of lipid homeostasis are prominent [114], caused by mutations of *NPC1* or *NPC2* [115,116]. Histologically, NPC is characterized by progressive loss of neurons, particularly Purkinje cells in the cerebellum, lipid storage, formation of meganeurites and ectopic dendrites as well as the presence of neurofibrillary tangles [117].

*NPC1a* null alleles in *Drosophila* die at an early larval stage, but feeding *NPC1a* mutants the steroid hormone (molting hormone) 20-hydroxyecdysone (20E) extends lifespan, suggesting that reduced ecdysone synthesis results from *NPC1a* loss. Feeding with excess cholesterol compounds extends lifespan further till adult stages. [118].

In another *Drosophila dnpc1a* model flies showed sterol accumulation as in human disease. By 7-dehydrocholesterol treatment life expectancy of *dnpc1a* mutants could be extended till adulthood. Brain morphology was unremarkable without any neurodegenerative changes [119].

In another study using the same *dnpc1a* mutants more extensive brain investigation revealed neuronal cholesterol deposits, accumulation of multilamellar bodies and age-dependent vacuolization. Age-dependent neurodegeneration, early lethality and movement disorders could all be completely rescued by neuronal and partially rescued by glial expression of wild-type *dNPC1a* transgene [120]. *Npc2a* mutants displayed a shorter life span, but did not show any brain vacuolization. TUNEL staining revealed neurons undergoing apoptosis [121].

Taken together, NPC can be modelled suitably in *Drosophila,* as cholesterol storage and neurodegenerative aspects of the disease are represented. On the other hand, one has to keep in mind two potential drawbacks of the invertebrate model when interpreting the results. Firstly *Drosophila* and other insects have redundant *npc1* and *npc2* genes (*npc1a, npc1b, npc2a, npc2b*), whereas mammals including humans only possess one *NPC1* and one *NPC2* gene. Functions of the other *npc* genes are not understood yet. Secondly, steroid actions in flies and humans are certainly different as flies cannot synthesize sterol, in particular the molting hormone 20E.

### 3.3. Ceroid lipofuscinoses

Ceroid lipofuscinoses are characterized by variable but mainly pediatric onset, vision loss, motor dysfunction, seizures and decline of intellectual capacities. [122]. Several causative genes, including *CLN2-3, 5–8, 10, PPT1* and *MFSD8* were identified [123,124]. Infantile neuronal ceroid lipofuscinosis (INCL), caused by loss of palmitoyl-protein thioesterase 1 (PPT1) on 1p32, shows brain atrophy, neuronal swelling, sudanophilic changes, granular osmiophilic deposits, lysosomal accumulation positive for acidic phophatase in neuronal and astrocytic cells, and rarefaction and shrinking of corticabasal and bulbar neurons [122].

*Palmitoyl-protein thioesterase 1 (Ppt1)* mutant flies have reduced life span and CNS-specific accumulation of autofluorescent storage material, which unlike human granular osmiophilic deposits were homogeneous in structure and composed of concentric layers of material [125]. The deposits may also be biochemically different as they could not be detected with lipophilic stains [125].

Targeted overexpression of *Ppt1* in the *Drosophila* visual system results in apoptotic neuronal cell loss, leading to misorganized ommatidia [126]. Performance of a gain-of-function modifier screen using enhancer-promoter lines could connect *Ppt1* function to synaptic vesicle cycling, endolysosomal trafficking and synaptic plasticity [127]. Observations in *Drosophila* have to be analyzed carefully as downregulation of *Ppt1* leads to accumulation of storage material, different from human GRODs without neurodegeneration und thus *Ppt1* in *Drosophila* might have a different function than *PPT1* in humans.

Although human disease is caused by loss of PPT1 and not overexpression, data from *Drosophila* studies reveal that misregulation of PPT1 may lead to neuronal cell loss and thus the correct titration of enzyme activity may be of importance. Nevertheless the possibility of modifier screens may reveal the physiological role of *Ppt1* in *Drosophila* and this may promote understanding human disease.

## 4. Tumors

### 4.1. Neurofibromatosis 1

Neurofibromatosis type 1 (NF1) is an autosomal dominantly inherited neurocutaneous disorder due to mutations in *NF1* on 17q11.2 [128]. NF1 is a common disease that mainly affects peripheral and central nervous system (neurofibromas, optic gliomas, astrocytomas, malignant peripheral nerve sheath tumors), the skin (café au lait spots, axillary and inguinal freckling), and may show further neuroendocrine/neuroectodermal tumors, hematopoietic tumors, osseous lesions, iris hamartomas and intellectual handicap [128].

Histologically dermal neurofibromas, well-circumscribed benign tumors composed of Schwann cells, as well as plexiform neurofibromas, producing diffuse enlargement of nerve trunks prevail [129]. Malignant peripheral nerve sheath tumors, highly aggressive tumors characterized by a herringbone pattern of cell growth, are localized within nerve fascicles but invade the adjacent soft tissues [129]. Gliomas are most often pilocytic astrocytomas within the optic nerve and bilateral growth is not uncommon in NF1 patients [129].

Manipulation of *NF1* gene was investigated during the last ten years in *Drosophila* showing that *neurofibromin* gene plays a role in tissue growth in all developmental stages [130], learning [131], circadian rest-activity rhythm [132], lifespan determination [133], Ras and cAMP interactions [133,134] and stress resistance [133]. Whether these findings will gain therapeutic consequences, for example by using antioxidative drugs, remains controversial. The main point that neurofibromatosis is characterized by appearance of multiple tumors in humans is not represented in all studies yet, thus modelling this disease in *Drosophila* remains disappointing.

### 4.2. Neurofibromatosis 2

Neurofibromatosis type 2 (NF2) primarily affects the nervous system, bilateral vestibular schwannomas being prominent. Schwannomas of other cranial nerves, meningiomas, ocular abnormalities, meningioangiomatosis, glial hamartomas and neurofibromas may also be present [128]. NF2 is an autosomal dominantly inherited neurocutaneous disorder due to mutations in *NF2* on 22q12.2 [128].

In *Drosophila*, *Merlin* mutations have been investigated and were shown to regulate cell growth and cell cycle [135]. It could be shown that *Merlin* is part of the *hippo* pathway [135]. Tumor formation in *Drosophila* could not be established by using *Merlin* mutations [136]. How these results could influence possible therapeutic options in NF2 patients remains to be elucidated.

### 4.3. Tuberous sclerosis

Tuberous sclerosis (TSC) is an autosomal dominant disease due to heterozygous mutations in *TSC1* on chromosome 9q34 or *TSC2* on chromosome 16p13 [137]. TSC is a neurocutaneous disorder characterized by brain abnormalities (cortical tubers, subependymal giant cell astrocytomas (SEGAs), subependymal glial nodules, seizures, mental retardation, autism and attention deficit-hyperactive disorders), kidney pathologies (angiomyolipomas, cysts and renal tumors) as well as rhabdomyomas of the heart [138].

Cortical tubers are strongly associated with the development of epilepsy, especially infantile spasms. They consist of giant cells, dysmorphic neurons, disrupted cortical lamination, gliosis and calcifications [139]. SEGAs are well-circumscribed, often calcified tumors with a mixed glioneuronal phenotype.

In *Drosophila, gigas (gig)* was identified as homolog of *TSC2*, leading to increase of cell size and imaginal discs as well as abnormal cell cycle progression [140]. While human SEGAs contain giant cells, corresponding D*rosophila* tissues in *gigas* mutated flies show hypertrophic changes but no distinct brain tumors.

### 4.4. Neuronal/neuroblastic tumors

Furthermore, several fly mutants interfering with asymmetric cell division of neuroblasts exhibit neuronal/neuroblastic tumors which are referred to as “hyperplastic” in case of preserved architecture such as *malignant brain tumor (l(3)mbt)* [141], or “neoplastic” with loss of architecture and invasion such as *brain tumor (brat)*, *raps, numb, pros* and *mira*, the latter four genes encoding for epithelial polarity proteins [142]. Results from these experiments show that maintenance of properly organized apical and basolateral domains is essential to prevent movement of cells out of an epithelium. Mutation of these genes may therefore contribute to metastasis. Different approaches have been pursued in *Drosophila* to model metastatic spread. Transplantation experiments with *brat, dlg* and *lgl* mutated brains into abdomens of adult flies showed that serial transplantations may lead to selection of more aggressive cell clones [143].

Modifier screens have been carried out and *Semaphorin-5c* and *apontic* were found to inhibit metastasis in a *lgl* mutant [144]. These kinds of screens will provide new insights into pathways involved in metastatic events.

Also reverse genetics can be applied to study invasion events as *Matrix metalloproteinase 1 (Mmp1)* known to promote invasion in humans was upregulated in metastasis models using *scribbled (scrib)* LOF mutations. Loss of *Mmp1* function by RNAi suppressed invasiveness [145].

Although some important anatomical features like vessels are lacking in *Drosophila* and polarity genes will be more important for carcinoma formation than for endogenous brain tumors, these models may help to understand a variety of basic cell biological features underlying tumor biology and may lead to new concepts in human tumor therapy. Remarkably, fly models of gliomas and medulloblastomas, the most common human brain tumors in adults and childhood, respectively, have not been published yet.

## 5. Epilepsy

Epilepsy is a neurological disorder characterized by bursts of abnormal electrical brain activity which can be classified into generalized seizures, focal (partial) seizures with or without secondary generalization and can be related to epilepsy syndromes which begin at a specific age and are associated with characteristic EEG patterns [146]. Epilepsies are classified into idiopathic forms that have no known cause except hereditary factors and symptomatic forms caused by brain lesions, such as malformations, tumors or asphyxia) [146]. Idiopathic epilepsy is predominantly associated with ion channel defects, including mutations in potassium-/sodium or calcium channel genes. Mutations of non-ion channel genes like *leucine-rich, glioma inactivated 1 gene (LGI1)* or *Aristaless related homeobox gene (ARX)* may also cause epilepsy syndromes [147,148]. Hippocampal sclerosis is the commonest neuropathological lesion identified in epilepsy patients. It is considered to represent both cause and consequence of seizures and can be classified according to varying degrees and localizations of neuronal cell loss [149].

Interestingly, electrical discharge patterns in flies resemble those described in kindling or after discharge stimulation protocols used in rodents [150,151]. By identifying the minimal voltage, which has to be applied to the fly brain in order to evoke a pattern of high-frequency neuronal firing followed by refractory inactivity, seizure susceptibility mutations have been detected. Among those were ethanolamine kinase*,* mitochondrial ribosomal proteins [152] as well as a number of channelopathy mutants such as *seizure, slowpoke, shaker* and *ether-a-go-go* [153,154]. Anticonvulsant drug screenings were performed and phenytoin as well as gabapentin were identified to be effective [155,156]. In mutant backgrounds several genetic modifiers of seizure activity could be detected [157–160], such as *top1,* which reduced seizure susceptibility. Pharmaceutical inhibition of topoisomerase I protein *(top1)* enzymatic activity was shown to reduce seizure sensitivity [161], which may lead to the discovery of new substances in human epilepsy therapy. Neuropathological investigations of seizure sensitive fly mutants revealed neuronal loss of varying degree but these could not be deduced to seizure activity alone but also resulted from metabolic defects of the respective mutations [162].

Future perspectives of epilepsy fly models could address additional human hereditary diseases in order to better understand the underlying genetic basis and to develop new therapy approaches by using large scale pharmacological screens.

## 6. Trauma

Traumatic CNS injury is common, comprising brain and spinal cord hemorrhage, contusion and diffuse axonal injury (DAI) leading to life long disability [163,164]. The injured adult central nervous system (CNS) inhibits axonal outgrowth thus limiting recovery from traumatic injury [163].

Histologically axonal injury is characterized by peri-wound sprouting without significant axonal growth beyond the lesion edge [165] and by presence of axonal bulbs (“retraction balls”) in the white matter, corpus callosum and brain stem [164]. These are caused by axonal perturbation with impairment of axoplasmic transport and swelling of the axon while the myelin sheath remains intact [164]. ß-Amyloid precursor-protein (ßAPP) undergoes fast axonal transport and therefore accumulates where axonal transport is impaired [164].

A *Drosophila* model for axonal injury and regeneration in the adult brain was established using microdissection trauma in a subpopulation of neurons in the adult brain, the small lateral neurons ventral (sLNv) [166]. After traumatic injury, wildtype sLNv proximal axonal stumps developed bulbar enlargements but failed to regenerate in a long-term, whole-brain explant culture. Regeneration could be enhanced by adult-specific overexpression of protein kinase A specifically in these neurons by PD-F-Gal4 driver line [166,167].

This is a very promising model as post traumatic changes resemble those seen in mammals including fragmentation of the distal stump and forming of retraction bulbs in the proximal stump [168], which is not due to culture conditions as morphology and function remain intact. Additionally cell-type specific screening may reveal molecules and genes involved in CNS axonal regeneration.

## 7. Conclusions

Taken together, as a wide variety of *Drosophila* models for neurodegenerative and metabolic brain diseases as well as epilepsy, tumors and trauma exist, it will now be necessary to compare similarities and differences of invertebrate and rodent models and human disease. Genetic tools will allow large modifier screens to reveal new pathways and interactions which could bring light into disease processes, which are not understood yet. Detection of genes modulating disease processes in the brain in *Drosophila* screens will have to be confirmed in higher model organisms to reach the goal of potential new medications for human diseases.
